# 3-(1-Benzofuran-2-yl)-1*H*-1,2,4-triazole-5(4*H*)-thione monohydrate

**DOI:** 10.1107/S1600536812025305

**Published:** 2012-06-13

**Authors:** Hoong-Kun Fun, Suhana Arshad, Nithinchandra, Balakrishna Kalluraya, Shobhitha Shetty

**Affiliations:** a X-ray Crystallography Unit, School of Physics, Universiti Sains Malaysia, 11800 USM, Penang, Malaysia; b Department of Studies in Chemistry, Mangalore University, Mangalagangothri 574 199, Karnataka, India

## Abstract

In the title hydrate, C_10_H_7_N_3_OS·H_2_O, the essentially planar benzofuran [maximum deviation = 0.006 (1) Å] and 4,5-dihydro-1*H*-1,2,4-triazole [maximum deviation = 0.007 (1) Å] rings form a dihedral angle of 11.67 (6)°. In the crystal, O—H⋯N, O—H⋯S, N—H⋯O and N—H⋯S hydrogen bonds link the mol­ecules into sheets lying parallel to the *bc* plane. Aromatic π–π stacking inter­actions [centroid–centroid distances = 3.5078 (8)–3.6113 (8) Å] are also observed.

## Related literature

For background to 1,2,4-triazoles, see: Shujuan *et al.* (2004 ▶); Clemons *et al.* (2004 ▶); Johnston (2002 ▶); Wei *et al.* (2007 ▶). For related structures, see: Jing *et al.* (2012 ▶); Fun *et al.* (2011 ▶); Abdel-Aziz *et al.* (2011 ▶). For stability of the temperature controller used in the data collection, see: Cosier & Glazer (1986 ▶).
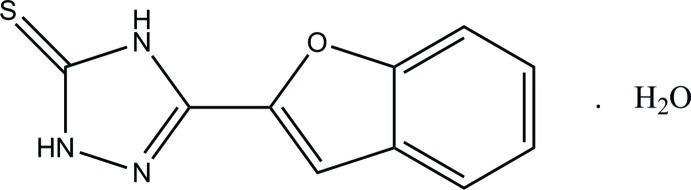


## Experimental

### 

#### Crystal data

C_10_H_7_N_3_OS·H_2_O
*M* *_r_* = 235.26
Monoclinic, 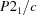

*a* = 7.1446 (1) Å
*b* = 8.8075 (1) Å
*c* = 17.3274 (2) Å
β = 111.942 (1)°
*V* = 1011.36 (2) Å^3^
*Z* = 4
Mo *K*α radiation
μ = 0.31 mm^−1^
*T* = 100 K
0.39 × 0.20 × 0.15 mm

#### Data collection

Bruker SMART APEXII CCD diffractometer
Absorption correction: multi-scan (*SADABS*; Bruker, 2009 ▶) *T*_min_ = 0.891, *T*_max_ = 0.955
19917 measured reflections
4162 independent reflections
3347 reflections with > *I* > 2σ(*I*)
*R*_int_ = 0.039

#### Refinement

*R*[*F* ^2^ > 2σ(*F* ^2^)] = 0.042
*wR*(*F* ^2^) = 0.108
*S* = 1.07
4162 reflections
153 parameters
H atoms treated by a mixture of independent and constrained refinement
Δρ_max_ = 0.61 e Å^−3^
Δρ_min_ = −0.31 e Å^−3^

### 

Data collection: *APEX2* (Bruker, 2009 ▶); cell refinement: *SAINT* (Bruker, 2009 ▶); data reduction: *SAINT*; program(s) used to solve structure: *SHELXTL* (Sheldrick, 2008 ▶); program(s) used to refine structure: *SHELXTL*; molecular graphics: *SHELXTL*; software used to prepare material for publication: *SHELXTL* and *PLATON* (Spek, 2009 ▶).

## Supplementary Material

Crystal structure: contains datablock(s) global, I. DOI: 10.1107/S1600536812025305/hb6837sup1.cif

Structure factors: contains datablock(s) I. DOI: 10.1107/S1600536812025305/hb6837Isup2.hkl

Supplementary material file. DOI: 10.1107/S1600536812025305/hb6837Isup3.cml

Additional supplementary materials:  crystallographic information; 3D view; checkCIF report

## Figures and Tables

**Table 1:** Hydrogen-bond geometry (Å, °)

*D*—H⋯*A*	*D*—H	H⋯*A*	*D*⋯*A*	*D*—H⋯*A*
O1*W*—H1*OW*⋯N2^i^	0.90	2.05	2.9135 (14)	160
O1*W*—H2*OW*⋯S1^ii^	0.82	2.46	3.2674 (11)	167
N1—H1*N*1⋯O1*W* ^iii^	0.90 (2)	1.81 (2)	2.7100 (14)	172.6 (19)
N3—H1*N*3⋯S1^iv^	0.846 (18)	2.498 (18)	3.3242 (10)	165.7 (16)

Symmetry codes: (i) 
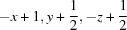
; (ii) 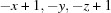; (iii) 
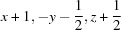
; (iv) 
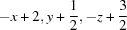
.

**Fig. 1.**
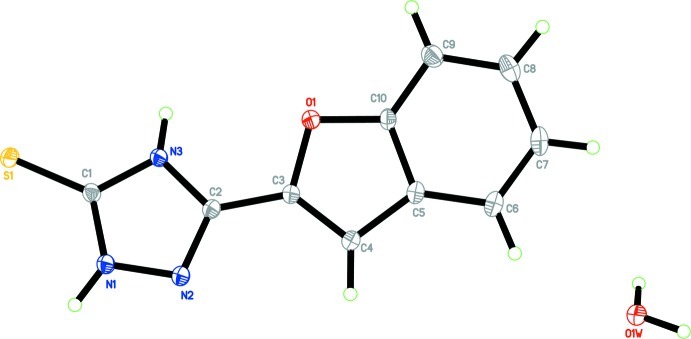
The molecular structure of the title compound, showing 50% probability displacement ellipsoids.

**Fig. 2.**
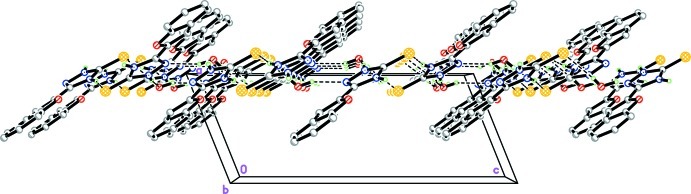
The crystal packing of the title compound, viewed down the b axis. The H atoms not involved in the intermolecular interactions (dashed lines) have been omitted for clarity.

## supplementary crystallographic information

### Comment

The 1,2,4-triazole nucleus has been incorporated into a wide variety of therapeutically interesting compounds. Several compounds containing 1,2,4-triazole rings are well known as drugs. For example, fluconazole is used as an antimicrobial drug (Shujuan *et al.*, 2004), whereas vorozole, letrozole and anastrozole are non-steroidal drugs used for the treatment of cancer (Clemons *et al.*, 2004) and loreclezole is used as an anticonvulsant (Johnston, 2002). Similarly substituted derivatives of triazole possess comprehensive bioactivities such as antimicrobial, anti-inflammatory, analgesic, antihypertensive, anticonvulsant and antiviral activities (Wei *et al.*, 2007). We now report the synthesis and crystal structure of the title compound.

The asymmetric unit of the title compound, (Fig. 1), consists of one 5-(1-Benzofuran-2-yl)-2,4-dihydro-3*H*-1,2,4-triazole-3-thione molecule and one water molecule. The benzofuran ring (O1/C3–C10) and the 4,5-dihydro -1*H*-1,2,4-triazole ring (N1–N3/C1/C2) are essentially planar with maximum deviations of 0.006 (1) Å at atom O1 and 0.007 (1) Å at atom N3, respectively. The dihedral angle between the benzofuran and 4,5-dihydro- 1*H*-1,2,4-triazole rings is 11.67 (6)°. Bond lengths and angles are within normal ranges and comparable to the related structures (Jing *et al.*, 2012; Fun *et al.*, 2011; Abdel-Aziz *et al.*, 2011).

The crystal packing is shown in Fig. 2. The molecules are linked *via* O1W—H1OW···N2, O1W—H2OW···S1, N1—H1N1···O1W and N3—H1N3···S1 hydrogen bonds (Table 1) into two-dimensional networks parallel to *bc*-plane. π–π interactions of *Cg*1···*Cg*1 = 3.6113 (8) Å (symmetry code: 1 - *x*, -*y*, 1 - *z*), *Cg*1···*Cg*2 = 3.5078 (8) Å (symmetry code: 2 - *x*, -*y*, 1 - *z*), *Cg*2···*Cg*3 = 3.5881 (8) Å (symmetry code: 1 - *x*, -*y*, 1 - *z*) and *Cg*3···*Cg*2 = 3.6056 (8) Å (symmetry code: 2 - *x*, -*y*, 1 - *z*) further stabilized the crystal structure [*Cg*1, *Cg*2 and *Cg*3 are the centroids of the O1/C3–C5/C10, N1–N3/C1/C2 and C5–C10 rings, respectively].

### Experimental

A mixture of 2-(1-benzofuran-2-ylcarbonyl)hydrazinecarbothioamide (0.01 mol) and 10% KOH (10 ml) was refluxed for 3 h. The mixture was cooled to room temperature and then neutralized by the gradual addition of glacial acetic acid. The solid product obtained was collected by filtration, washed with ethanol and dried. It was then recrystallized using ethanol. Yellow blocks of the title compound were obtained by slow evaporation of the ethanolic solution.

### Refinement

O– and N-bound H atoms were located from a difference Fourier map. O-bound H atoms were fixed at their found positions (O–H = 0.8961 and 0.8208 Å), with *U*_iso_(H) = 1.5 *U*_eq_(O), whereas N-bound H atoms was refined freely [N–H = 0.844 (18) and 0.90 (2) Å]. The remaining H atoms were positioned geometrically [C–H = 0.93 Å] and refined using a riding model with *U*_iso_(H) = 1.2 *U*_eq_(C). In the final refinement, one outlier (1 1 1) was omitted.

### Crystal data

**Table:**

C_10_H_7_N_3_OS·H_2_O	*F*(000) = 488
*M**_r_* = 235.26	*D*_x_ = 1.545 Mg m^−^^3^
Monoclinic, *P*2_1_/*c*	Mo *K*α radiation, λ = 0.71073 Å
Hall symbol: -P 2ybc	Cell parameters from 6273 reflections
*a* = 7.1446 (1) Å	θ = 2.5–33.4°
*b* = 8.8075 (1) Å	µ = 0.31 mm^−^^1^
*c* = 17.3274 (2) Å	*T* = 100 K
β = 111.942 (1)°	Block, yellow
*V* = 1011.36 (2) Å^3^	0.39 × 0.20 × 0.15 mm
*Z* = 4	

### Data collection

**Table:**

Bruker SMART APEXII CCD diffractometer	4162 independent reflections
Radiation source: fine-focus sealed tube	3347 reflections with > *I* > 2σ(*I*)
Graphite monochromator	*R*_int_ = 0.039
φ and ω scans	θ_max_ = 34.2°, θ_min_ = 2.5°
Absorption correction: multi-scan (*SADABS*; Bruker, 2009)	*h* = −11→11
*T*_min_ = 0.891, *T*_max_ = 0.955	*k* = −13→13
19917 measured reflections	*l* = −26→27

### Refinement

**Table:**

Refinement on *F*^2^	Primary atom site location: structure-invariant direct methods
Least-squares matrix: full	Secondary atom site location: difference Fourier map
*R*[*F*^2^ > 2σ(*F*^2^)] = 0.042	Hydrogen site location: inferred from neighbouring sites
*wR*(*F*^2^) = 0.108	H atoms treated by a mixture of independent and constrained refinement
*S* = 1.07	*w* = 1/[σ^2^(*F*_o_^2^) + (0.0491*P*)^2^ + 0.4347*P*] where *P* = (*F*_o_^2^ + 2*F*_c_^2^)/3
4162 reflections	(Δ/σ)_max_ = 0.001
153 parameters	Δρ_max_ = 0.61 e Å^−^^3^
0 restraints	Δρ_min_ = −0.31 e Å^−^^3^

### Special details

**Table:**

Experimental. The crystal was placed in the cold stream of an Oxford Cryosystems Cobra open-flow nitrogen cryostat (Cosier & Glazer, 1986) operating at 100.0 (1) K.
Geometry. All e.s.d.'s (except the e.s.d. in the dihedral angle between two l.s. planes) are estimated using the full covariance matrix. The cell e.s.d.'s are taken into account individually in the estimation of e.s.d.'s in distances, angles and torsion angles; correlations between e.s.d.'s in cell parameters are only used when they are defined by crystal symmetry. An approximate (isotropic) treatment of cell e.s.d.'s is used for estimating e.s.d.'s involving l.s. planes.
Refinement. Refinement of *F*^2^ against ALL reflections. The weighted *R*-factor *wR* and goodness of fit *S* are based on *F*^2^, conventional *R*-factors *R* are based on *F*, with *F* set to zero for negative *F*^2^. The threshold expression of *F*^2^ > σ(*F*^2^) is used only for calculating *R*-factors(gt) *etc*. and is not relevant to the choice of reflections for refinement. *R*-factors based on *F*^2^ are statistically about twice as large as those based on *F*, and *R*- factors based on ALL data will be even larger.

### Fractional atomic coordinates and isotropic or equivalent isotropic displacement parameters (Å^2^)

**Table:**

	*x*	*y*	*z*	*U*_iso_*/*U*_eq_	
S1	1.16013 (5)	−0.27662 (3)	0.789110 (18)	0.01410 (8)	
O1	0.76589 (13)	0.12211 (10)	0.53014 (5)	0.01383 (17)	
N1	1.01801 (16)	−0.33637 (12)	0.62307 (6)	0.01333 (19)	
N2	0.91410 (16)	−0.27263 (11)	0.54676 (6)	0.01371 (19)	
N3	0.95013 (15)	−0.11026 (11)	0.64872 (6)	0.01228 (18)	
C1	1.04179 (18)	−0.24126 (13)	0.68643 (7)	0.0121 (2)	
C2	0.87285 (17)	−0.13492 (13)	0.56444 (7)	0.0120 (2)	
C3	0.76153 (17)	−0.02501 (13)	0.50237 (7)	0.0122 (2)	
C4	0.65217 (18)	−0.03918 (13)	0.41967 (7)	0.0139 (2)	
H4A	0.6284	−0.1277	0.3882	0.017*	
C5	0.58061 (17)	0.11092 (14)	0.39077 (7)	0.0134 (2)	
C6	0.46309 (19)	0.17499 (15)	0.31360 (8)	0.0169 (2)	
H6A	0.4126	0.1157	0.2658	0.020*	
C7	0.42492 (19)	0.32943 (16)	0.31110 (8)	0.0185 (2)	
H7A	0.3474	0.3741	0.2606	0.022*	
C8	0.5000 (2)	0.42034 (15)	0.38265 (9)	0.0193 (2)	
H8A	0.4710	0.5236	0.3784	0.023*	
C9	0.6168 (2)	0.35953 (14)	0.45977 (8)	0.0175 (2)	
H9A	0.6675	0.4189	0.5075	0.021*	
C10	0.65281 (18)	0.20498 (13)	0.46092 (7)	0.0128 (2)	
O1W	0.12342 (14)	0.13102 (10)	0.11923 (6)	0.01697 (18)	
H1OW	0.0827	0.1677	0.0673	0.025*	
H2OW	0.0538	0.1810	0.1383	0.025*	
H1N1	1.062 (3)	−0.433 (2)	0.6261 (12)	0.032 (5)*	
H1N3	0.933 (3)	−0.032 (2)	0.6736 (11)	0.016 (4)*	

### Atomic displacement parameters (Å^2^)

**Table:**

	*U*^11^	*U*^22^	*U*^33^	*U*^12^	*U*^13^	*U*^23^
S1	0.01807 (14)	0.01305 (13)	0.00963 (13)	0.00052 (9)	0.00341 (10)	0.00123 (9)
O1	0.0168 (4)	0.0119 (4)	0.0113 (4)	0.0021 (3)	0.0035 (3)	−0.0001 (3)
N1	0.0172 (5)	0.0122 (4)	0.0099 (4)	0.0022 (3)	0.0043 (3)	0.0014 (3)
N2	0.0167 (5)	0.0130 (4)	0.0103 (4)	0.0017 (3)	0.0038 (3)	0.0009 (3)
N3	0.0158 (4)	0.0110 (4)	0.0098 (4)	0.0021 (3)	0.0044 (3)	0.0005 (3)
C1	0.0137 (5)	0.0112 (4)	0.0118 (5)	0.0005 (4)	0.0051 (4)	0.0013 (4)
C2	0.0132 (5)	0.0126 (5)	0.0100 (5)	0.0005 (4)	0.0040 (4)	0.0004 (4)
C3	0.0130 (5)	0.0121 (5)	0.0108 (5)	0.0008 (4)	0.0036 (4)	0.0007 (4)
C4	0.0152 (5)	0.0132 (5)	0.0115 (5)	0.0006 (4)	0.0027 (4)	−0.0001 (4)
C5	0.0123 (5)	0.0159 (5)	0.0113 (5)	0.0007 (4)	0.0038 (4)	0.0024 (4)
C6	0.0156 (5)	0.0214 (6)	0.0120 (5)	0.0006 (4)	0.0030 (4)	0.0029 (4)
C7	0.0153 (5)	0.0224 (6)	0.0168 (6)	0.0041 (4)	0.0049 (4)	0.0085 (5)
C8	0.0187 (5)	0.0166 (5)	0.0236 (6)	0.0048 (4)	0.0090 (5)	0.0061 (5)
C9	0.0200 (6)	0.0147 (5)	0.0182 (6)	0.0029 (4)	0.0077 (4)	0.0010 (4)
C10	0.0134 (5)	0.0135 (5)	0.0108 (5)	0.0018 (4)	0.0037 (4)	0.0026 (4)
O1W	0.0225 (4)	0.0144 (4)	0.0136 (4)	0.0006 (3)	0.0064 (3)	0.0009 (3)

### Geometric parameters (Å, º)

**Table:**

S1—C1	1.6892 (12)	C4—H4A	0.9300
O1—C10	1.3784 (14)	C5—C10	1.4003 (17)
O1—C3	1.3785 (14)	C5—C6	1.4045 (17)
N1—C1	1.3403 (16)	C6—C7	1.3848 (19)
N1—N2	1.3715 (14)	C6—H6A	0.9300
N1—H1N1	0.90 (2)	C7—C8	1.403 (2)
N2—C2	1.3112 (15)	C7—H7A	0.9300
N3—C1	1.3664 (15)	C8—C9	1.3914 (18)
N3—C2	1.3718 (15)	C8—H8A	0.9300
N3—H1N3	0.844 (18)	C9—C10	1.3840 (17)
C2—C3	1.4448 (16)	C9—H9A	0.9300
C3—C4	1.3575 (16)	O1W—H1OW	0.8961
C4—C5	1.4382 (16)	O1W—H2OW	0.8208
			
C10—O1—C3	105.34 (9)	C10—C5—C6	118.94 (11)
C1—N1—N2	113.03 (10)	C10—C5—C4	105.90 (10)
C1—N1—H1N1	127.4 (13)	C6—C5—C4	135.16 (12)
N2—N1—H1N1	119.6 (13)	C7—C6—C5	117.72 (12)
C2—N2—N1	103.96 (10)	C7—C6—H6A	121.1
C1—N3—C2	107.80 (10)	C5—C6—H6A	121.1
C1—N3—H1N3	125.3 (12)	C6—C7—C8	121.81 (12)
C2—N3—H1N3	126.5 (12)	C6—C7—H7A	119.1
N1—C1—N3	104.16 (10)	C8—C7—H7A	119.1
N1—C1—S1	127.44 (9)	C9—C8—C7	121.54 (12)
N3—C1—S1	128.40 (9)	C9—C8—H8A	119.2
N2—C2—N3	111.04 (10)	C7—C8—H8A	119.2
N2—C2—C3	123.72 (11)	C10—C9—C8	115.71 (12)
N3—C2—C3	125.24 (10)	C10—C9—H9A	122.1
C4—C3—O1	112.58 (10)	C8—C9—H9A	122.1
C4—C3—C2	131.59 (11)	O1—C10—C9	125.35 (11)
O1—C3—C2	115.82 (10)	O1—C10—C5	110.37 (10)
C3—C4—C5	105.81 (10)	C9—C10—C5	124.28 (11)
C3—C4—H4A	127.1	H1OW—O1W—H2OW	101.1
C5—C4—H4A	127.1		
			
C1—N1—N2—C2	−0.29 (14)	C2—C3—C4—C5	−178.79 (12)
N2—N1—C1—N3	−0.49 (13)	C3—C4—C5—C10	−0.85 (13)
N2—N1—C1—S1	179.94 (9)	C3—C4—C5—C6	179.54 (14)
C2—N3—C1—N1	1.06 (13)	C10—C5—C6—C7	0.09 (18)
C2—N3—C1—S1	−179.38 (9)	C4—C5—C6—C7	179.66 (13)
N1—N2—C2—N3	0.98 (13)	C5—C6—C7—C8	0.00 (19)
N1—N2—C2—C3	−179.39 (11)	C6—C7—C8—C9	0.0 (2)
C1—N3—C2—N2	−1.34 (14)	C7—C8—C9—C10	−0.17 (19)
C1—N3—C2—C3	179.04 (11)	C3—O1—C10—C9	−179.75 (12)
C10—O1—C3—C4	0.01 (13)	C3—O1—C10—C5	−0.58 (13)
C10—O1—C3—C2	179.45 (10)	C8—C9—C10—O1	179.33 (11)
N2—C2—C3—C4	11.6 (2)	C8—C9—C10—C5	0.28 (19)
N3—C2—C3—C4	−168.79 (13)	C6—C5—C10—O1	−179.42 (10)
N2—C2—C3—O1	−167.68 (11)	C4—C5—C10—O1	0.90 (13)
N3—C2—C3—O1	11.90 (17)	C6—C5—C10—C9	−0.24 (19)
O1—C3—C4—C5	0.54 (14)	C4—C5—C10—C9	−179.92 (12)

### Hydrogen-bond geometry (Å, º)

**Table:**

*D*—H···*A*	*D*—H	H···*A*	*D*···*A*	*D*—H···*A*
O1*W*—H1*OW*···N2^i^	0.90	2.05	2.9135 (14)	160
O1*W*—H2*OW*···S1^ii^	0.82	2.46	3.2674 (11)	167
N1—H1*N*1···O1*W*^iii^	0.90 (2)	1.81 (2)	2.7100 (14)	172.6 (19)
N3—H1*N*3···S1^iv^	0.846 (18)	2.498 (18)	3.3242 (10)	165.7 (16)

Symmetry codes: (i) −*x*+1, *y*+1/2, −*z*+1/2; (ii) −*x*+1, −*y*, −*z*+1; (iii) *x*+1, −*y*−1/2, *z*+1/2; (iv) −*x*+2, *y*+1/2, −*z*+3/2.
